# A Larger Income and Smaller Debt

**Published:** 1920-04-03

**Authors:** 


### ENCOURAGING PROGRESS AT CHESTER.

### A Larger Income and Smaller Debt.

The report of the Chester Royal Infirmary for 1919 shows that after meeting the year's expenses, the debt has been reduced from £19,324 to £11,351. The ordinary expenditure was £16,770. Among the receipts, which merit attention, are the following: Special Thanksgiving Appeal (Peace), £5,574; donations, £2,053; Hospital Saturday Committee and workmen's collections, £1,086, and £600 received too late for inclusion; Pound Day (with £1,000 from the Duke of Westminster), £1,117; Victory Ball, £952; legacies, £4,396.

Mr. E. M. Sneyd Kynnersley, the Chairman of the Board of Management, described at the annual meeting the reorganisation which had followed the withdrawal of the soldiers. Wards had been opened for eye and other special departments, an orthopædic clinic established, and definite provision was to be made for paying patients. A new children's ward had also been opened on the second floor of the renovated old wing, with a roof balcony adjoining, and commanding an uninterrupted view of the Welsh hills. On the ward being allotted to the children, two friends of the hospital—Dr. J. George Taylor, hon. physician, and Mr. W. Heathcote Williams—had offered to take it over and to hand it back decorated, furnished and equipped. The offer was gratefully accepted by the Board, and the outcome is a ward as attractive as art can make it, with every modern appliance and convenience.

### An Artistic Ward

The plans were prepared with Mr. C. Ogilvie Campbell, of Chester, as architect, after visits to well-known hospitals, and many ideas were conceived and developed as the work progressed. The walls and ceiling are enamelled, with a high dado surmounted by a 10-in. panel and a deep frieze, both of nursery patterns. New fire-grates have nursery rhyme panels above, and over the balcony door are leaded light window-panels. New electric fittings and additional heating in the sanitary annexe have been installed, and twenty latest-pattern cots. A complete equipment of bedding and linen, instruments, nursery and all other furniture and domestic utensils, and the furnishing of the sister's room, are included in the gift. It may be added that arrangements have been made with local authorities for the admission under the Maternity and Child Welfare scheme of children under five years of age. The ward was dedicated by the Bishop of Chester (Dr. H. L. Paget) in the presence of a large gathering.

A suggestion has been made, and is supported by the local press, that Chester should raise an endowment fund of £10,000 for the ward, in addition to a memorial cross, which is to be placed in the centre of the city. The donors of the ward have offered, if this suggestion is adopted, to regard their gift as part of the war memorial, so that the War Memorial of Chester may be a memorial cross, and the provision and endowment of the children's ward of the infirmary.

The hours of duty and salaries of the nursing staff have been revised. The day staff has been granted one day in seven off duty, and night nurses have three consecutive night's off duty per month. Salaries of ward sisters have been raised to £52-£5-£62; and of nurses, to first year, £18; second year, £22; third year, £26; fourth year, £35.

### The Growth of Employers' Subscriptions.

Turning to the future, the Chairman was able to announce that already this year legacies amounting to over £6,000 had been notified, and a gift of £1,000 received from the Joint Committee of the B.R.C.S. and St. John. He also mentioned the continued increase of the Income of the Hospital Saturday Committee, and the receipt from employers of gifts equivalent to their employees' collections. Since his speech, one amount of £700 has been so received.

